# Synchronous small bowel neuroendocrine tumour, colonic adenocarcinoma, and non-Hodgkin lymphoma: a rare triad of primary malignancies

**DOI:** 10.1093/jscr/rjaf970

**Published:** 2025-12-04

**Authors:** Niamh Keating, Tim Harding, Leya Motala, Faraz Khan, Conor Shields

**Affiliations:** Royal College of Surgeons in Ireland, 123 St Stephen’s Green, Dublin D02 YN77, Ireland; Department of Colorectal Surgery, Mater Misericordiae University Hospital, Eccles St, Phibsborough, Dublin D07 R2WY, Ireland; Royal College of Surgeons in Ireland, 123 St Stephen’s Green, Dublin D02 YN77, Ireland; Department of Colorectal Surgery, Mater Misericordiae University Hospital, Eccles St, Phibsborough, Dublin D07 R2WY, Ireland; Department of Pathology, Mater Misericordiae University Hospital, Eccles St, Phibsborough, Dublin D07 R2WY, Ireland; Department of Colorectal Surgery, Mater Misericordiae University Hospital, Eccles St, Phibsborough, Dublin D07 R2WY, Ireland; Department of Colorectal Surgery, Mater Misericordiae University Hospital, Eccles St, Phibsborough, Dublin D07 R2WY, Ireland

**Keywords:** synchronous malignancies, neuroendocrine tumour, colorectal cancer, lymphoma

## Abstract

A man in his 70s presented to the Emergency Department with a history of progressive dyspnoea and was found to be anaemic. Endoscopic evaluation revealed a caecal lesion, confirmed histologically as a moderately differentiated adenocarcinoma. Staging computed-tomography imaging demonstrated widespread lymphadenopathy including pelvic, mediastinal and axillary regions, that appeared unlikely to be related to the colonic primary. Surgical resection of the right colon and a palpable axillary node was performed, during which a separate jejunal lesion was incidentally identified and resected. Histopathological analysis confirmed three distinct neoplasms: colonic adenocarcinoma, a small bowel neuroendocrine tumour, and non-Hodgkin lymphoma. Immunohistochemical and molecular profiling confirmed independent origins with no shared clonal relationship. The following case highlights the diagnostic and therapeutic challenges posed by synchronous multiple primary malignancies, particularly across divergent tissue lineages. Accurate histopathological distinction and multidisciplinary coordination are essential to guide management, prioritize treatment, and optimize patient outcomes in such exceptional presentations.

## Introduction

Multiple primary malignancies (MPM) are defined as two or more histologically distinct cancers arising in the same individual. According to the International Agency for Research on Cancer, tumours are classified as synchronous when diagnosed concurrently or within six months of one-another, and metachronous when diagnosed beyond this interval [1].

The reported incidence of MPM ranges from 2% to 17% of cancer patients. The apparent rise in incidence of MPM across the literature to date reflects not only improved cancer survivorship but also advances in cross-sectional imaging, endoscopic-screening, and post-treatment surveillance strategies [2].

Interestingly, neuroendocrine tumours (NET) appear disproportionately represented among patients with MPM, suggesting a possible biological predisposition.

This association was first reported by Pearson and Fitzgerald in 1944, who identified second primary malignancies in 23% of carcinoid tumour autopsies [3]. Subsequent institutional and population-based studies have consistently supported this observation [4, 5]. Nonetheless, only isolated cases of triple synchronous malignancies involving NET have been described [6, 7].

Most reports of MPM document the coexistence of two tumours. Triple primary malignancies are significantly less common, affecting <1% of cancer patients [8]. Furthermore, the vast majority are metachronous, rendering synchronous triple malignancies an exceptionally rare occurrence. We describe the case of a patient diagnosed concurrently with a small-bowel NET, colonic adenocarcinoma, and non-Hodgkin B-cell lymphoma, representing a highly unusual triad across distinct tissue lineages.

## Case report

A 77 year-old man attended the Emergency Department with a history of progressive, exertional dyspnoea over 2–3 months. Prior to this he maintained an active lifestyle, walking 1–2 km per day with a good functional baseline. He had a history of hypertension, coronary artery disease, and atrial fibrillation, for which he was taking a direct oral anticoagulant. Surgical history included right total hip replacement. He was a nonsmoker, did not drink alcohol, and had no personal or family history of malignancy.

Initial laboratory investigation demonstrated microcytic anaemia (haemoglobin 8.9 g/dL) and urgent gastroscopy and colonoscopy were arranged. Gastroscopy was unremarkable, however; colonoscopy identified an ulcerated caecal lesion which was biopsied. Histopathological analysis confirmed the presence of an invasive caecal adenocarcinoma.

A contrast-enhanced computed-tomography (CT) scan of the thorax, abdomen, and pelvis was subsequently undertaken for staging. This demonstrated the caecal tumour but no regional ileocaecal lymphadenopathy or distant metastases. However, multiple enlarged lymph-nodes were noted in the pelvic, hilar, mediastinal, and axillary regions, which were considered unrelated to the caecal primary.

The case was reviewed at the Gastrointestinal Surgery Multidisciplinary Meeting. The consensus recommendation was to proceed with surgical resection of the caecal malignancy and to further investigate possible underlying haematological malignancy by way of excisional biopsy of a palpable, enlarged lymph node.

**Figure 1:**
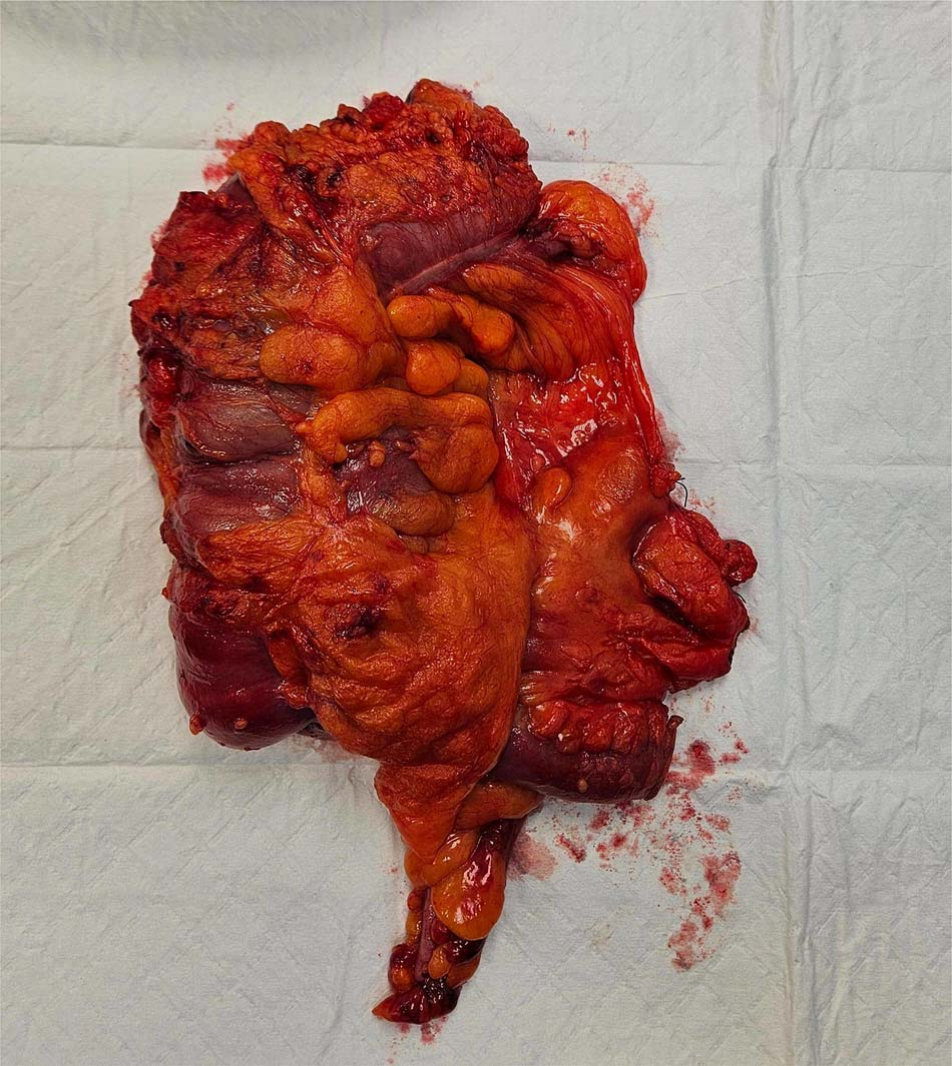
Right hemicolectomy.

**Figure 2:**
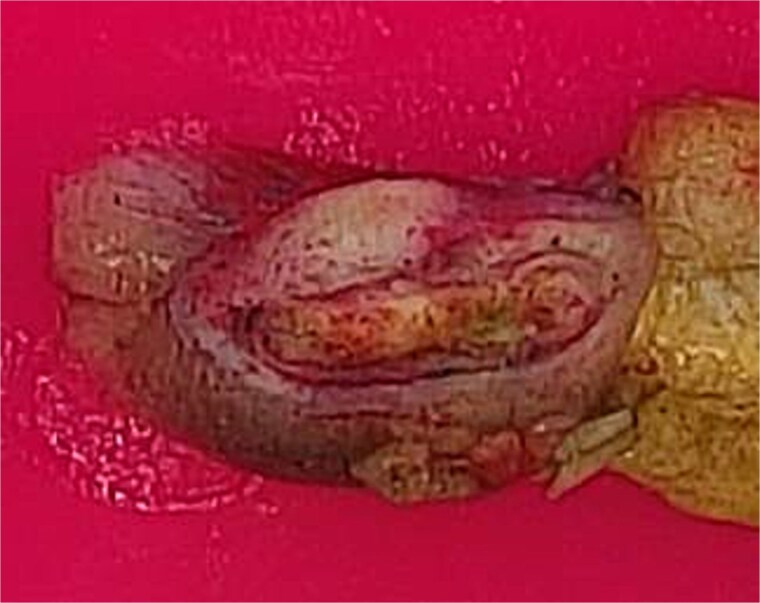
Resected small bowel segment with attached mesentery.

**Figure 3:**
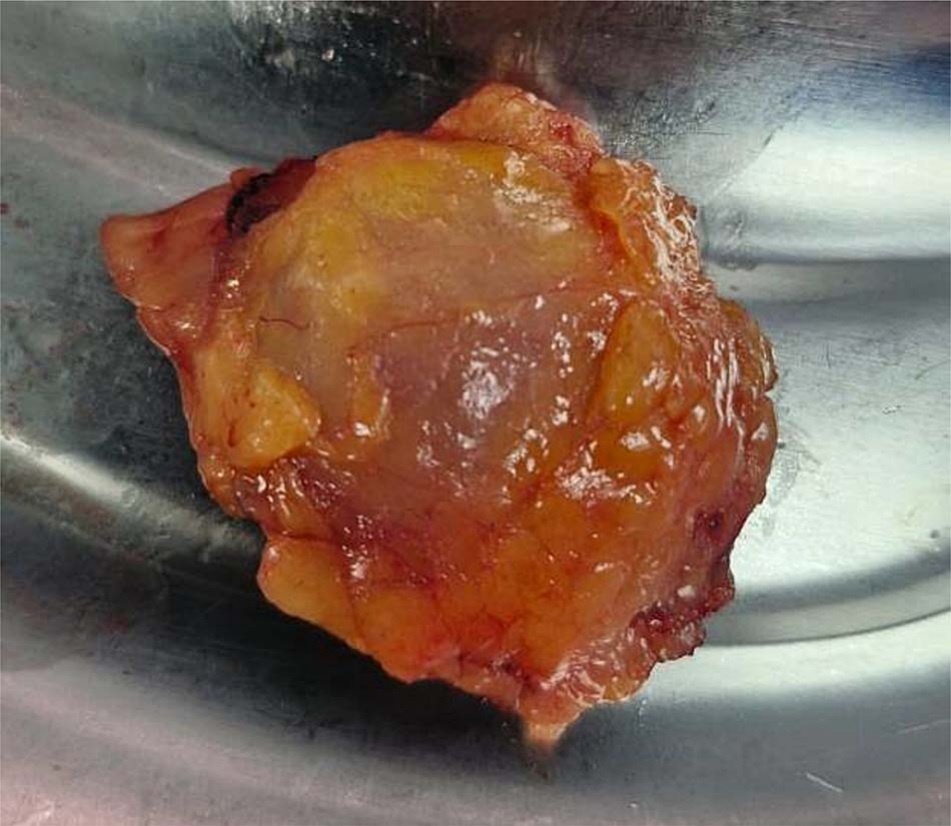
Left axillary lymph node.

The patient underwent laparoscopic-assisted right hemicolectomy and ileocaecal anastomosis (Fig. 1). Intraoperatively, a small lesion was incidentally identified arising from the small bowel ~1m proximal to the caecum. This segment of small bowel was resected *en bloc*, and intestinal continuity restored with a side-to-side anastomosis (Fig. 2). Subsequently, a palpable axillary lymph-node, previously identified on CT imaging, was excised (Fig. 3).

**Figure 4:**
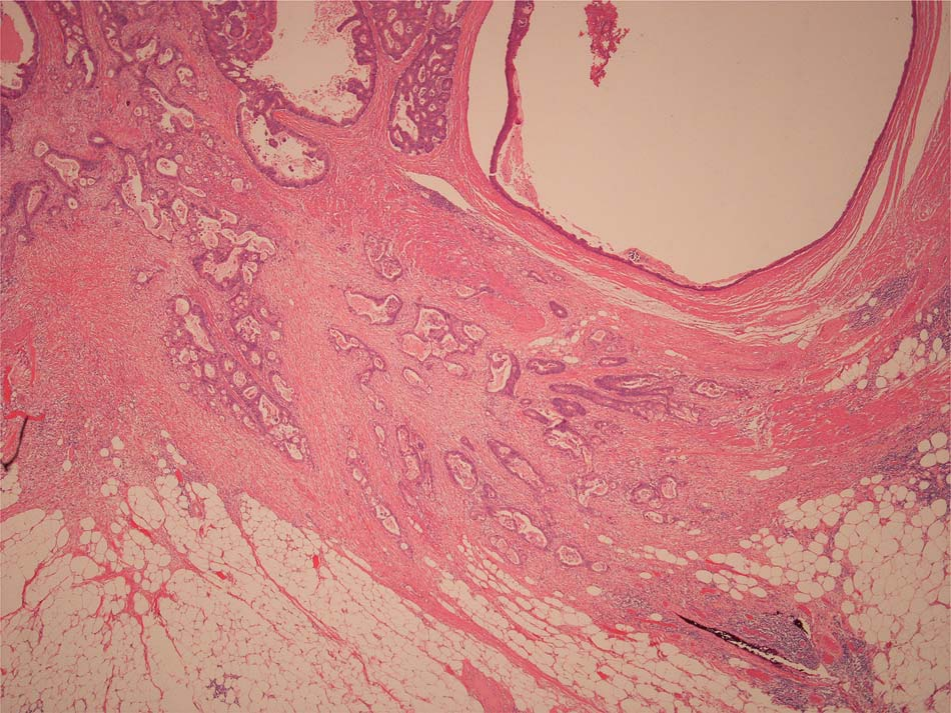
Low power view of colonic adenocarcinoma invading muscularis propria and pericolonic fat.

**Figure 5:**
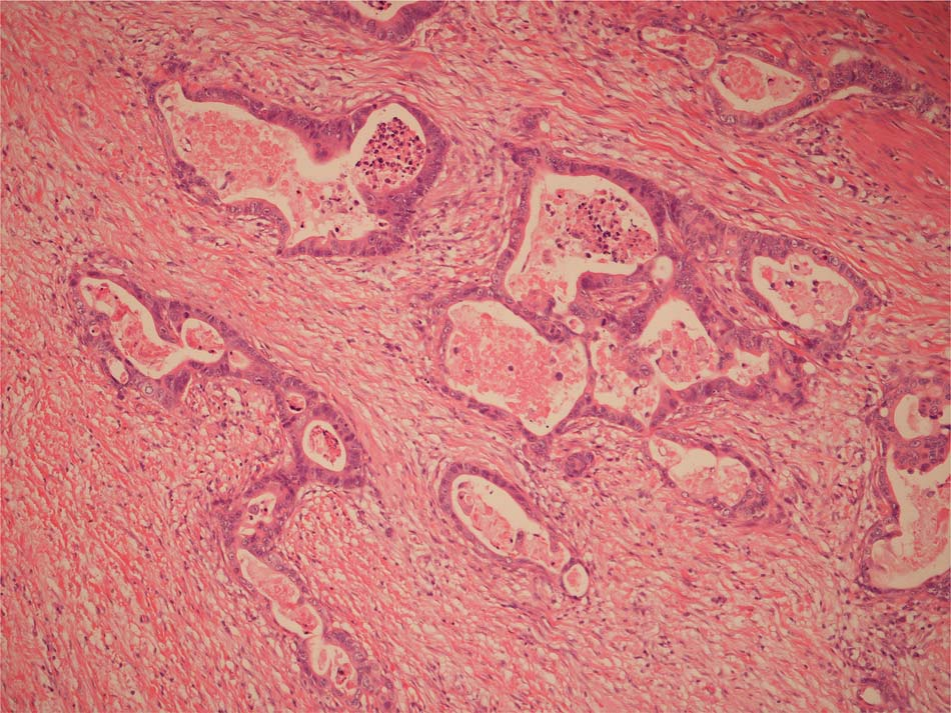
High power view of colonic adenocarcinoma showing complex glandular structures lined by epithelial cells with high grade atypia, and lumina containing necrotic debris, invading beyond the mucosa.

**Figure 6:**
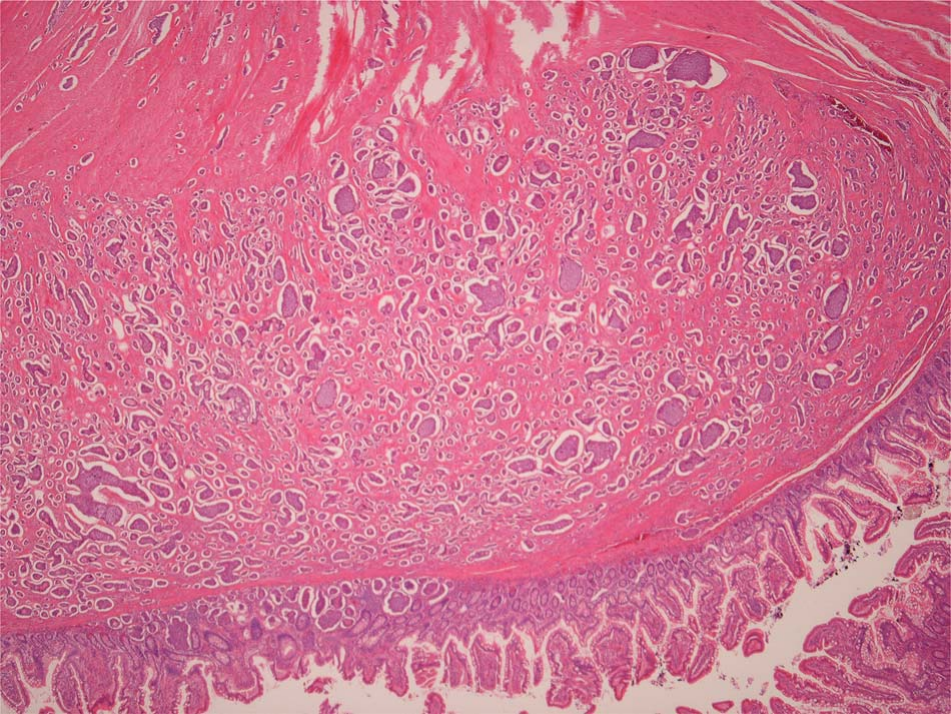
Low power view of submucosal neuroendocrine tumour.

**Figure 7:**
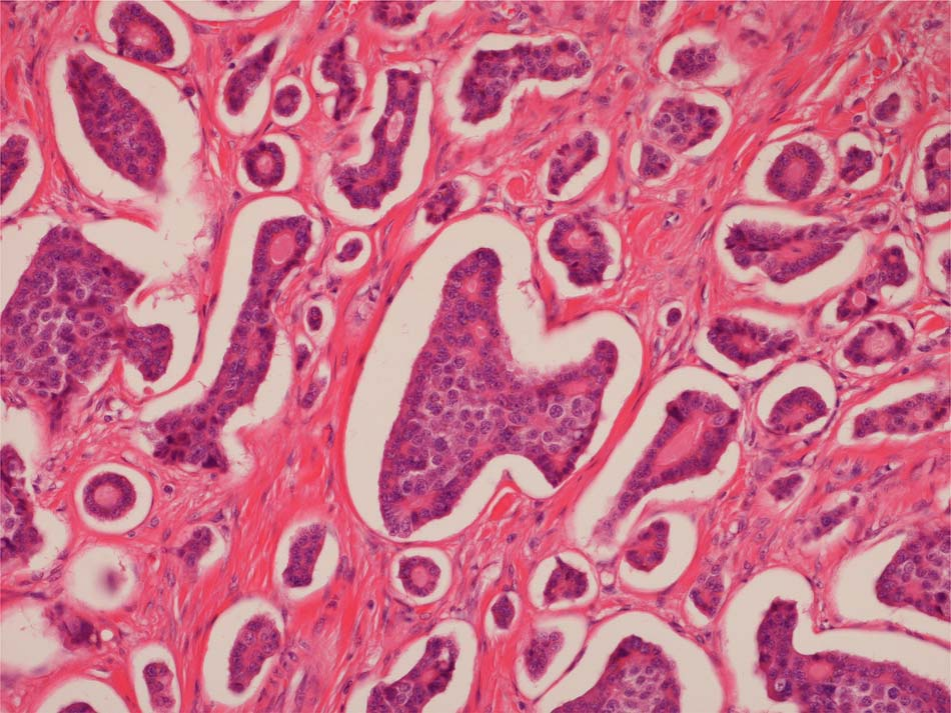
High power view of well-differentiated neuroendocrine tumour showing nests of monotonous cells with round nuclei and “salt and pepper” chromatin.

**Figure 8:**
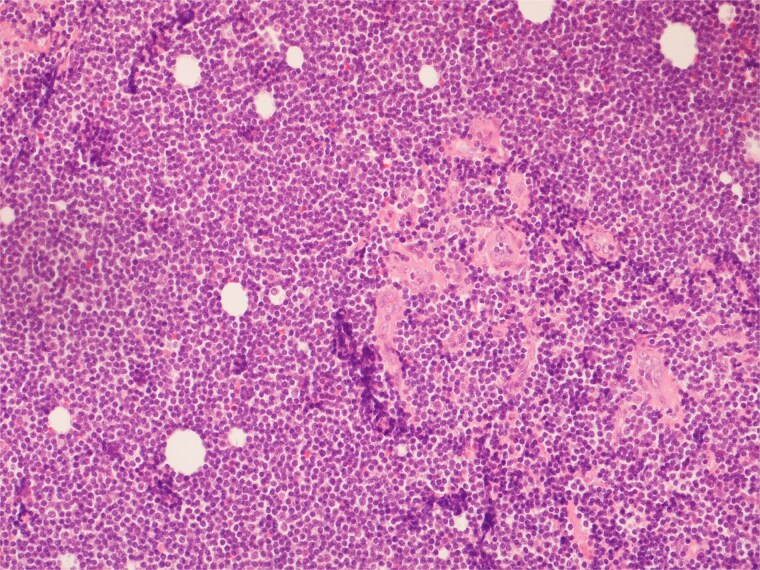
High power view showing a proliferation of monotonous small lymphoid cells.

**Figure 9:**
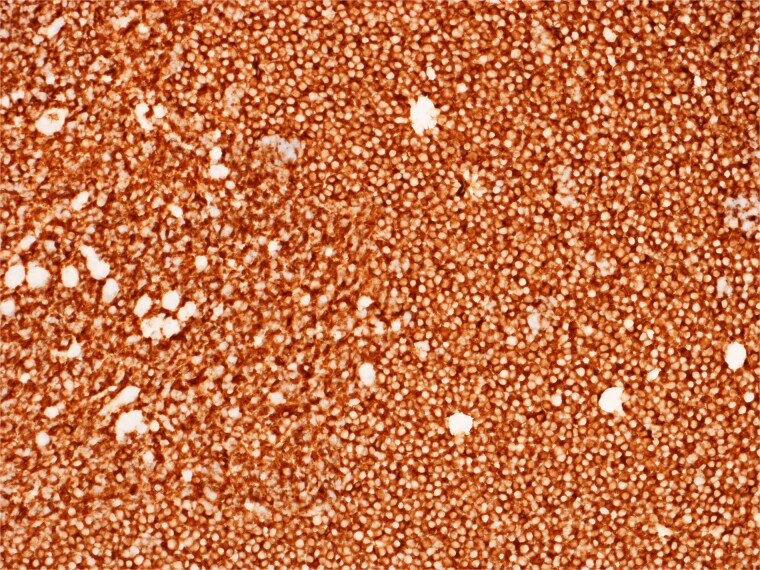
Diffusely and strongly positive CD20 staining of neoplastic lymphoid cells.

Histopathological examination of surgical specimens revealed three distinct neoplasms: (i) invasive colonic adenocarcinoma (Figs 4 and 5), (ii) jejunal NET (Figs 6 and 7), and (iii) small-lymphocytic lymphoma (Figs 8 and 9). Morphological assessment supported by immunohistochemical profiling confirmed each represented an independent primary, with no evidence of a shared clonal origin. The findings were subsequently reviewed at the Multidisciplinary Meeting alongside Oncology and Haematology specialists, and adjuvant single-agent chemotherapy was initiated, with planned surveillance of the indolent lymphoma.

## Discussion

The widely accepted diagnostic criteria of MPM were first proposed by Warren and Gates: each tumour must represent a definite malignancy, each must be histopathologically distinct and the possibility of one representing a metastasis of the other must be excluded [9].

The association between NET and MPM is increasingly recognized, although the mechanisms underpinning this relationship remain poorly understood. One proposed explanation suggests that bioactive trophic factors secreted from neuroendocrine cells—such as gastrin and glucagon—may act as mitogens, stimulating proliferation in adjacent tissues, and promoting neoplastic transformation [10]. Supporting this, various other malignancies have been shown to express receptors for these bioactive substances [11, 12]. However, such a mechanism is unlikely to account for cases in which an NET is detected years after a previous malignancy.

The Field-Effect theory offers an alternative hypothesis, proposing that repeated exposure of the gastrointestinal tract to an array of environmental carcinogens can induce neoplastic transformation across multiple cell types [4]. While this may explain the coexistence of gastrointestinal NET with other gastrointestinal malignancies, it does not account for tumours arising in anatomically and embryologically distinct systems.

Genetic predisposition and familial cancer syndromes including Lynch syndrome and Multiple Endocrine Neoplasia are well-established contributors to MPM [13]. Environmental and host-related factors, such as smoking, immunodeficiency, and prior chemotherapy/radiotherapy have also been implicated [2]. In this case, the patient had no identifiable hereditary or lifestyle risk factors. Furthermore, the three neoplasms originated from embryologically distinct lineages: hindgut-derived colonic epithelium, midgut-derived jejunal neuroendocrine cells, and mesodermal lymphoid tissue—and shared no pathogenic mutations on molecular analysis. Collectively, these findings support a sporadic origin.

MPM diagnosis presents considerable clinical challenges. Overlapping, nonspecific symptoms may obscure additional tumours, particularly when one lesion dominates the clinical picture. Radiological abnormalities may be misinterpreted as metastases, leading to under-recognition of synchronous primaries. Finally, diagnostic bias may arise once an initial malignancy is identified, reducing the likelihood of investigating additional lesions with equal scrutiny [14].

There are no dedicated guidelines specifically tailored for management of MPM. Treatment is generally directed toward the most aggressive or clinically significant tumour first [15]. In some instances, a single therapeutic modality—such as surgery or systemic therapy—may address multiple primaries; however, staged or combination approaches are often required. The coexistence of synchronous malignancies may also limit systemic therapy options due to overlapping toxicities or contraindications [2].

Optimal management of MPM demands multidisciplinary collaboration among surgical, medical, and radiation oncologists. Although rare, cases such as this highlight the importance of maintaining a high index of suspicion for additional malignancies, even after initial cancer diagnosis. Advances in molecular profiling may, in future, clarify whether these unusual constellations arise through chance coincidence or shared oncogenic mechanisms.
